# Synthesis and Application of Ferroelectric Poly(Vinylidene Fluoride-co-Trifluoroethylene) Films using Electrophoretic Deposition

**DOI:** 10.1038/srep36176

**Published:** 2016-11-02

**Authors:** Jeongjae Ryu, Kwangsoo No, Yeontae Kim, Eugene Park, Seungbum Hong

**Affiliations:** 1 Department of Materials Science and Engineering, Korea Advanced Institute of Science and Technology, Daejeon 34141, Republic of Korea; 2 Materials and Energy Science and Engineering, Nelson Mandela African Institute of Science and Technology, Arusha 447, Tanzania; 3 Materials Science Division, Argonne National Laboratory, Lemont, Illinois 60439, USA

## Abstract

In this study, we investigated the deposition kinetics of polyvinylidene fluoride copolymerized with trifluoroethylene (P(VDF-TrFE)) particles on stainless steel substrates during the electrophoretic deposition (EPD) process. The effect of applied voltage and deposition time on the structure and ferroelectric property of the P(VDF-TrFE) films was studied in detail. A method of repeated EPD and heat treatment above melting point were employed to fabricate crack-free P(VDF-TrFE) thick films. This method enabled us to fabricate P(VDF-TrFE) films with variable thicknesses. The morphology of the obtained films was investigated by scanning electron microscopy (SEM), and the formation of β-phase was confirmed by X-ray diffraction (XRD) and Fourier transform infrared (FTIR) spectroscopy. P(VDF-TrFE) films prepared with various thicknesses showed remnant polarization (P_r_) of around 4 μC/cm^2^. To demonstrate the applicability of our processing recipe to complex structures, we fabricated a spring-type energy harvester by depositing P(VDF-TrFE) films on stainless steel springs using EPD process. Our preliminary results show that an electrophoretic deposition can be applied to produce high-quality P(VDF-TrFE) films on planar as well as three-dimensional (3-D) substrates.

Many research groups have studied ferroelectric polymers due to their light weight, high flexibility, nontoxicity, and easy processability^1^,^2^,^3^,^4^,^5^,^6^,^7^,^8^,^9^. Among them, polyvinylidene fluoride (PVDF) and its copolymer with trifluoroethylene (P(VDF-TrFE)) are considered to be candidate materials for nonvolatile memory, sensors, actuators, solar cells, and energy harvesting systems^1^,^2^,^3^,^4^,^5^ because of their highly compact structure, relatively large permanent dipole moment, and high chemical stability. P(VDF-TrFE) films are typically fabricated by spin coating^6^, Langmuir-Blodgett deposition^7^, dip coating^8^ and solution casting^9^. These methods are based on chemical solution deposition. They are useful for smooth and flat thin films, but they have limited application for very large area and complex-shaped structures, such as foam and porous substrates.

Meanwhile, electrophoretic deposition (EPD) has advantages over other methods of preparing P(VDF-TrFE) thick films on substrates with various shapes because the deposition rate of EPD is high, and the viscosity of the suspension is low^10^,^11^. More specifically, EPD is a colloidal process, which comprises two steps. The first step is to induce motion of charged particles in suspension under the influence of an applied electric field. The second step is to deposit the charged particles onto oppositely charged electrodes. Recently, EPD has attracted much attention because of its versatility, easy process, and need for a simple equipment, such as a power supply^10^,^11^. This method has been used to deposit metal^12^, polymer^13^, ceramic^14^, and biomaterial^15^ particles in the range from nanometers to a few micrometers on conducting substrates or even conducting substrates covered with a dielectric material^16^. In addition, it has been used to deposit two kinds of materials on substrates simultaneously^17^. Many different types of ferroelectric ceramics have been widely used in the EPD process, such as Pb(Zr,Ti)O_3_^18^,^19^,^20^,^21^, K_0.5_Na_0.5_NbO_3_^22^, BaTiO_3_^23^,^24^,^25^ and BiFeO_3_^26^. Moreover, the deposition of ferroelectric ceramics on complex substrates, such as helical structures^27^, individual fibers, fiber mats, and nichrome wires^28^ has been achieved using EPD.

However, there has been no report on the kinetics of EPD for the growth of ferroelectric polymer P(VDF-TrFE) films as well as its applications to complex structures. Although Foster *et al*. demonstrated P(VDF-TrFE) films fabricated by EPD on a silicon substrate^29^, the detailed kinetics involved in the EPD of P(VDF-TrFE) particles, such as deposited weight dependence on applied voltage and deposition time, has not been reported. In this study, we investigated the kinetics of P(VDF-TrFE) particles on stainless steel substrates and fabricated crack-free P(VDF-TrFE) thick films with thicknesses in the range of 21.4–34.8 μm by means of repeated EPD and heat treatment. Furthermore, we characterized the ferroelectric properties of P(VDF-TrFE) films as a function of the film thickness using XRD, FTIR, and polarization-electric field hysteresis loop measurements. Finally, we demonstrated that the P(VDF-TrFE) films could be coated on a complex structured substrate using repeated EPD and heat treatment.

## Results and Discussion

**Figure 1:**
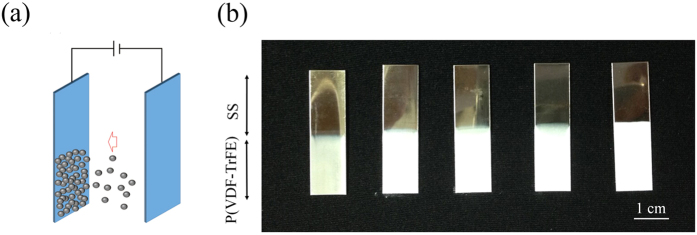
(**a**) Schematic illustration of the electrophoretic deposition process of P(VDF-TrFE) particles on a stainless steel substrate. (**b**) Photographic images of the substrates covered with P(VDF-TrFE) particles by the electrophoretic deposition process with applied voltages from 10 to 50 V for 2 min.

The schematic illustration of the experimental apparatus shown in Fig. 1(a) depicts the transport and adhesion of P(VDF-TrFE) particles with negative zeta potential to the anode driven by the external electric field. We carried out P(VDF-TrFE) deposition on the stainless steel substrates for various times and at various voltages with a constant distance between the electrodes. The samples of P(VDF-TrFE) deposited using EPD are shown in Fig. 1(b). We observed that the anode was covered with P(VDF-TrFE) particles because the applied electric field pushes the electrophoretic motion of the negatively charged particles toward the anode. We further confirmed that the P(VDF-TrFE) suspension has a negative zeta potential of −26.94 mV by zeta potential analyzer. In addition, we could deposit thicker films on the stainless steel substrates as we increased the voltage to the anode from 10 to 50 V.

**Figure 2:**
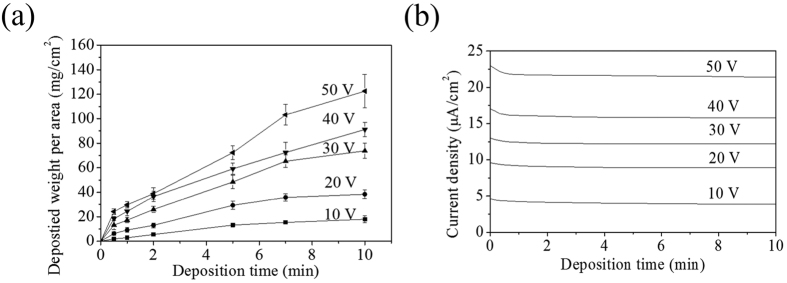
Plots of deposited weight per area as (**a**) a function of deposition time with different applied voltages and (**b**) current density versus deposition time at different applied voltages.

We measured the deposited weight per area (mg cm^−2^) as a function of the deposition time and the applied voltage during electrophoretic deposition of P(VDF-TrFE) particles in isopropyl alcohol. We found that the deposited weight per area increased with the deposition time as shown in Fig. 2(a). We also found that the deposited weight per area increased almost linearly with increasing applied voltage. Additionally, we observed that the deposition rate of P(VDF-TrFE) particles increased as the applied voltage increased from 10 to 50 V. However, if all other parameters were kept constant, the deposition rate of P(VDF-TrFE) particles, which is defined by the slope of the curve in Fig. 2(a), decreased with increasing deposition time except for a few points in 20 and 50 V. Figure 2(b) shows the current density as a function of the deposition time and the applied voltage. The current density slightly decreased as the deposition time increased and saturated at 80–90% of its initial value at 10 min. It has been often observed by other research groups^30^,^31^ that both the deposition rate and the current density decrease as the deposition time increases, which has been attributed to the higher resistance of the films as the film thickness increases or the concentration of the particles in solution decreases. Therefore, we speculate that the initial decreasing part of the current resulted from the combined contribution of the increase in the film resistance and the decrease in the concentration. However, the latter part where the current stayed constant can be explained only if the porosity remains high so that the current component related to the charge flow through the porous region contributes to the output current. Moreover, we observed that the deposition was close to zero when the applied voltage was 5 V even though we increased the deposition time up to 1 h. Based on this observation, we infer that there is a threshold electric field between 5 V/cm and 10 V/cm, below which no or very little electrophoretic deposition of P(VDF-TrFE) particles occurs on stainless steel substrates. This threshold value is different from that (32 V/cm) reported in the literature by Foster *et al*.^29^, where they used silicon as the substrate, indicating strong dependence of the threshold field on the substrate.

**Figure 3:**
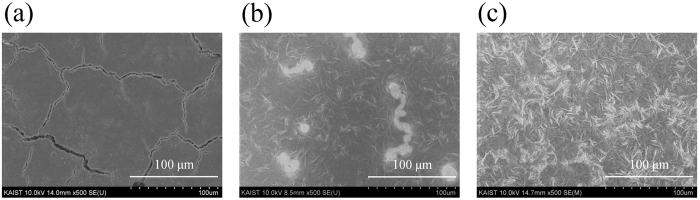
(**a**) SEM images of P(VDF-TrFE) films deposited on stainless steel substrates at 30 V for 2 min before heat treatment, (**b**) after heat treatment and (**c**) after second deposition and heat treatment.

Figure 3(a) shows an SEM image of P(VDF-TrFE) particles deposited on a stainless steel substrate at 30 V for 2 min. We observed that the P(VDF-TrFE) films contained microcracks over their surfaces. When a P(VDF-TrFE) film dries, tensile stress develops due the capillary forces, and the stress is relieved by the formation and propagation of cracks^32^. In addition, compared to the applied voltages at 10 and 20 V, larger sized cracks were formed at 30, 40, and 50 V (Supplementary Information, Figs S1–S4). It should be noted that the P(VDF-TrFE) film in Fig. 3(a) peeled from the substrates when a weak force was applied, indicating poor adhesion between the coating and the substrate. To enhance the adhesion and remove the microcracks, we annealed the film at 150 °C for 6 h, which was above the melting point of 147 °C measured by differential scanning calorimetry (DSC). The film color changed from white to transparent. Furthermore, we found that the adhesion of the P(VDF-TrFE) film to the substrate was enhanced. However, we observed that part of the substrate was not covered completely due to the shrinkage of the film, as shown in Fig. 3(b). To overcome this problem, both deposition and heat treatment were repeated on the same substrate twice^29^,^33^. Current densities during the 2^nd^ deposition were down to a very low level than those during the 1^st^ deposition because the voltage drop existed across the P(VDF-TrFE) film formed by the 1^st^ deposition and heat treatment (see Supplementary Information, Fig. S5). Figure 3(c) shows an SEM image of P(VDF-TrFE) film after repeated deposition and heat treatment. It can be seen that the whole substrate was covered with P(VDF-TrFE) film.

**Figure 4:**
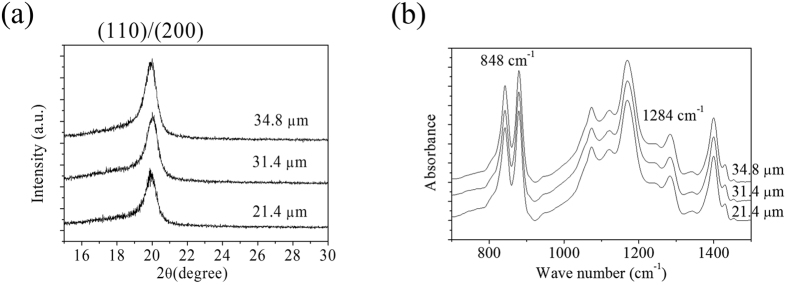
(**a**) XRD patterns and (**b**) FTIR spectra of P(VDF-TrFE) films with different thickness.

Crack-free films with thickness of 21.4, 31.4, and 34.8 μm were obtained by repeated deposition at 30, 40, and 50 V for 2 min and heat treatment, respectively. To check the ferroelectric phase formation in the films, we conducted X-ray diffraction (XRD) and Fourier transform infrared (FTIR) analysis on P(VDF-TrFE) films with various thicknesses. It is well known that the prominent peak at 2θ = 19.7° is the sum reflections of (110) and (200) planes of the crystalline β phase^34^. In Fig. 4(a), we found that the peak exists on the films ranging from 21.4 to 34.8 μm and the position of the peak remains the same regardless of the film thickness, indicative of similar inter-chain spacing for all the films fabricated by EPD in this study. Figure 4(b) shows the FTIR results (from 1500 to 700 cm^−1^) of P(VDF-TrFE) films with various thicknesses in the range of 21.4–34.8 μm. There are six bands associated with the three axes of the crystal of the P(VDF-TrFE)^35^,^36^,^37^. The peaks at 890 cm^−1^ and 1184 cm^−1^ correspond to the a-axis, the 848 cm^−1^ and 1284 cm^−1^ peaks indicate the b-axis, and the 1078 cm^−1^ and 1400 cm^−1^ peaks are associated with the c-axis. Among them, the characteristic absorbance band of 848 cm^−1^ represents the symmetric vibration of CF_2_(ν_s_CF_2_) coupled with the symmetric stretching vibration of C-C(ν_s_CC), and that of 1284 cm^−1^ represents ν_s_CF_2_ coupled with ν_s_CC and the bending vibration of C-C-C(δCCC)^35^,^36^,^37^. Those at 848 cm^−1^ and 1284 cm^−1^ are associated with trans sequences longer than TTTT and TTT, respectively^35^,^36^,^37^. As shown in Fig. 4(b), the 1284 cm^−1^ and 848 cm^−1^ bands were nearly at the same position and the peak intensities were similar regardless of film thickness from 21.4 to 34.8 μm, indicating that the β phase crystal orientation does not change with thickness. Therefore, based on the FTIR and XRD results, we confirmed that the P(VDF-TrFE) films were composed of the crystalline β-phase with the same molecular chain structure regardless of film thickness from 21.4 to 34.8 μm.

**Figure 5:**
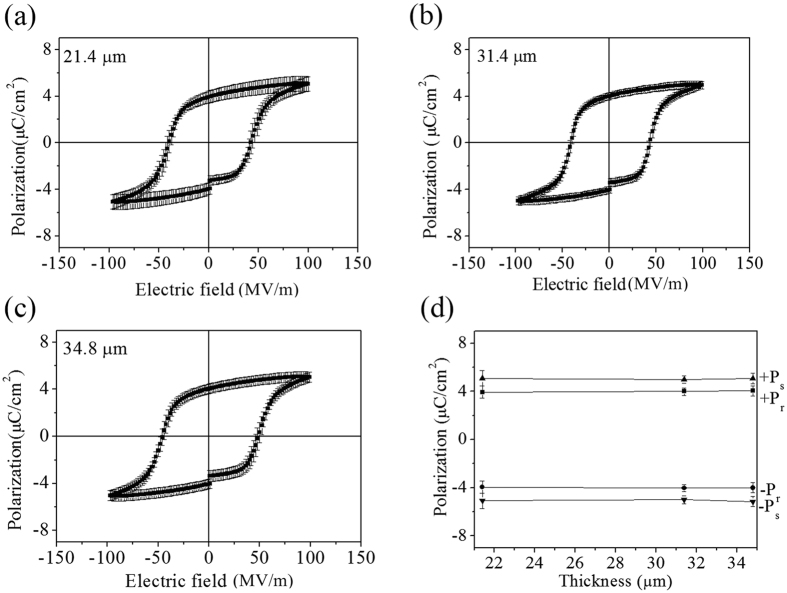
Polarization-electric field hysteresis of (**a**) 21.4 μm, (**b**) 31.4 μm, and (**c**) 34.8 μm thick P(VDF-TrFE) films measured at 100 Hz. (**d**) Saturation polarization and remnant polarization versus thickness in P(VDF-TrFE) films.

The polarization-electric field (P-E) hysteresis loop was measured at 100 Hz to examine the ferroelectric properties of the P(VDF-TrFE) films fabricated by EPD. We also investigated whether there is any change in ferroelectric properties as a function of the film thickness. For the characterization of ferroelectric properties, we fabricated a metal-ferroelectric-metal (MFM) capacitor structure. Pt top electrodes were deposited on P(VDF-TrFE) films by sputtering using a shadow mask with holes of 400 μm in diameter. For the 21.4 μm thick film, the P-E loops showed a saturation polarization (P_s_) of 5.1 μC/cm^2^ and remnant polarization (P_r_) of 3.9 μC/cm^2^ with a coercive field E_c_ of approximately 42 MV/m. The P_r_, P_s_ and E_c_ of the 31.4 μm-thick film in a saturated curve were approximately 4.0 μC/cm^2^, 5.0 μC/cm^2^, and 42 MV/m, respectively. We found P_s_ of 5.1 μC/cm^2^, P_r_ of 4.0 μC/cm^2^, and E_c_ of 47 MV/m for the 34.8 μm thick film. As shown in Fig. 5(d), the ferroelectric properties remained the same over a large range of thickness, and this trend is supported by the findings shown in Fig. 4, where the crystal and molecular structures remained the same regardless of the film thickness. Therefore, we developed a simple and cost effective process to fabricate thick P(VDF-TrFE) films with uniform ferroelectric properties, which provides a large processing window in terms of film thickness.

**Figure 6:**
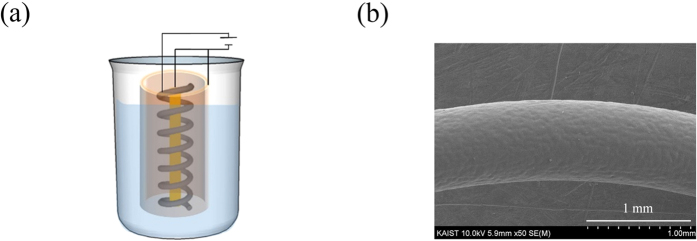
(**a**) Schematic illustration of the electrophoretic deposition on a spring. (**b**) SEM image of P(VDF-TrFE) films coated on the spring using repeated EPD and heat treatment.

We applied repeated EPD and heat treatment above the melting point to the fabrication of P(VDF-TrFE) films on a stainless steel spring. The spring was 0.89 mm in wire diameter, 15.03 mm in outer spring diameter and 40.28 mm in length. The spring had 6 turns. The counter electrodes consisted of an outer cylindrical electrode and an inner cylindrical electrode as shown Fig. 6(a). The distance between the spring and each part of the counter electrodes was 3 mm. The applied voltage was 10 V and the deposition time was 1 min. Figure 6(b) is an SEM image showing the crack-free surface of the P(VDF-TrFE) films on the spring after EPD and heat treatment twice. The thickness of P(VDF-TrFE) films on the spring was 25 μm.

**Figure 7:**
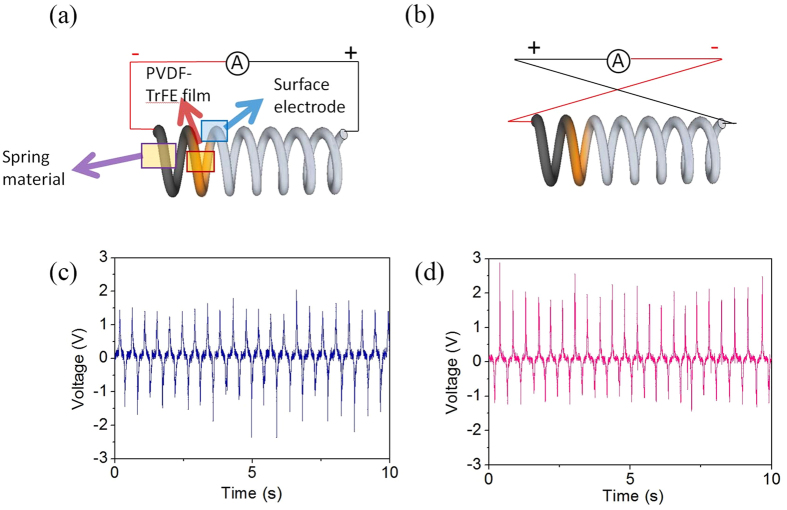
Schematic illustration of (**a**) forward connection and (**b**) reverse connection of SPEH. The output voltage of SPEH under (**c**) forward connection and (**d**) reverse connection during pressing and release.

We also measured the output voltage signal from the spring-type piezoelectric energy harvester (SPEH) as the SPEH was periodically pressed and released. To uniformly coat the top of the spring with the Al electrode by thermal evaporation, we repeated the coating process 3 times, rotating the spring by 120° about its long axis^38^. Teflon tape was used to mask both end of the spring to prevent two opposite electrodes from electrically being connected during the coating process. To enhance the output voltage of the SPEH, the poling process is an essential step, in which P(VDF-TrFE) films are poled under the electric field. The poling process was performed at 100 °C with a bias voltage of 500 V for 1 h using the power supply. After the poling process, the output voltage of the SPEH approximately enhanced by 4.4 times, as shown in the Supplementary Information, Fig. S6. When the output voltage was measured, the displacement of the spring from its resting position was about 10 mm. In order to prove that the output voltage was the genuine piezoelectric signal, we compared the output voltage of the SPEH under the forward and the reverse connections. The average output voltage under the forward connection and the reverse connection was 1.71 V and 1.79 V, respectively (Fig. 7). In the reverse connection, the polarity of the output voltage was reversed and the amplitude of the signal was almost identical with the forward connection. The results indicated that the measured output voltage was the true signal generated from the SPEH.

## Conclusions

In this study, ferroelectric polymer P(VDF-TrFE) films on stainless steel substrate were fabricated by electrophoretic deposition (EPD) in isopropyl alcohol. We found that the amount of the particles deposited on the substrates increased with the applied voltage and the deposition time. By means of repeated deposition and heat treatment above melting point, we could solve the surface coverage and adhesion problems. Furthermore, XRD and FTIR studies confirmed the presence of β-phase in the P(VDF-TrFE) films prepared by EPD. Finally, the P(VDF-TrFE) films prepared with various thicknesses showed a remnant polarization (P_r_) of around 4 μC/cm^2^, indicative of uniform ferroelectric properties over the thickness range from 21.4 to 34.8 μm. We applied this method to the fabrication of P(VDF-TrFE) films on a spring and confirmed that SPEH can generate electrical energy by mechanical pressure. Therefore, we believe that electrophoretic deposition is a promising method to produce high-quality P(VDF-TrFE) thick films with low cost and simple apparatus.

## Methods

### Electrophoretic deposition of P(VDF-TrFE)

The powder used in this study was 80/20 P(VDF-TrFE) with an average particle size of 270 nm (Measurement Specialties, Inc.). To prepare the P(VDF-TrFE) suspension, 0.6 g P(VDF-TrFE) powder was dispersed in 60 mL of isopropyl alcohol by ultrasonication for 60 min to break up agglomeration. Stainless steel substrates with dimensions of 1 mm × 10 mm × 40 mm were used for both the cathode and the anode. Prior to the EPD experiment, the stainless steel substrates were cleaned with acetone, ethanol, and distilled water in an ultrasonic bath for 10 min, respectively. The deposition area was fixed as 10 mm × 20 mm, and the electrodes were separated by 10 mm. The EPD parameters, such as the applied voltage and the deposition time, were varied from 10 to 50 V and from 30 s to 10 min, respectively. A constant electric voltage was applied by a power supply (Glasman High Voltage, Inc). The obtained electrophoretic deposits were dried in air at room temperature for 100 min and weighed by an electric balance with an accuracy of 0.0001 g. The current density was recorded by a digital multimeter attached in series during the EPD process.

### Characterization

The zeta potential of the suspension was estimated from the electrophoretic mobility measured using a zeta potential analyzer (Otsuka Electronics, ELS-Z2). The surface morphology was observed by scanning electron microscopy (SEM, S-4800, Hitachi). The film thickness was measured by an Alpha-step 500 surface profilometer (Tencor instruments). The formation of the ferroelectric β-phase in P(VDF-TrFE) films was confirmed by X-ray diffraction (XRD) (Rigaku D/Max-2500) and Fourier transform infrared (FTIR) spectroscopy in the attenuated total reflection mode (Bruker, IFS66V/S & HYPERION 3000) with a scanning resolution of 2 cm^−1^. The polarization-electric field (P-E) hysteresis loop was measured using a virtual-ground technique using an RT66A test system (Precision, RT-66A) equipped with a high-voltage amplifier (Trek Inc.). The output voltage of the spring type energy harvesters was monitored by a digital oscilloscope (DPO3034, Tektronix, Inc.). Input resistance and capacitance at the probe tip were 10 MΩ and <8 pF, respectively.

## Additional Information

**How to cite this article**: Ryu, J. *et al*. Synthesis and Application of Ferroelectric Poly(Vinylidene Fluoride-co-Trifluoroethylene) Films using Electrophoretic Deposition. *Sci. Rep.* **6**, 36176; doi: 10.1038/srep36176 (2016).

**Publisher’s note:** Springer Nature remains neutral with regard to jurisdictional claims in published maps and institutional affiliations.

## Supplementary Material

Supplementary Information
