# Supplementary material for: Synthesis and Application of Ferroelectric Poly(Vinylidene Fluoride-co-Trifluoroethylene) Films using Electrophoretic Deposition
Source: Sci Rep. 2016 Nov 2;6:36176. doi: 10.1038/srep36176 (PMC5090457; doi:10.1038/srep36176)
Supplement: Supplementary Information [file srep36176-s1.pdf]

Supplementary Information for

Synthesis and Application of Ferroelectric Poly(Vinylidene-Co-Trifluoroethylene) Films using Electrophoretic Deposition

Jeongjae Ryu[1], Kwangsoo No[1,*], Yeontae Kim[1], Eugene Park[2], and Seungbum Hong[1,3,*]

[*]Corresponding authors: ksno@kaist.ac.kr, hong@anl.gov

[1] *Department of Materials Science and Engineering, Korea Advanced Institute of Science and Technology, Daejeon 34141, Republic of Korea*

[2]*Materials and Energy Science and Engineering, Nelson Mandela African Institute of Science and Technology, Arusha 447, Tanzania*

[3]*Materials Science Division, Argonne National Laboratory, Lemont, Illinois 60439, USA*

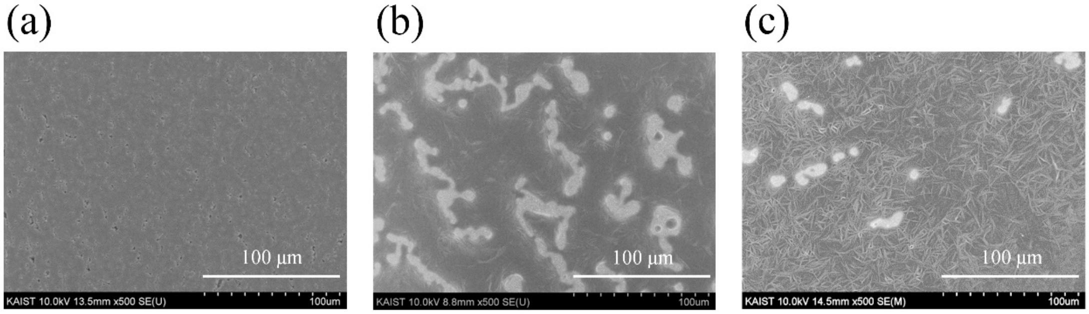

Fig. S1. SEM images of P(VDF-TrFE) films deposited on stainless steel substrates at 10 V for 2 min (a) before heat treatment, (b) after heat treatment and (c) after second deposition and heat treatment.

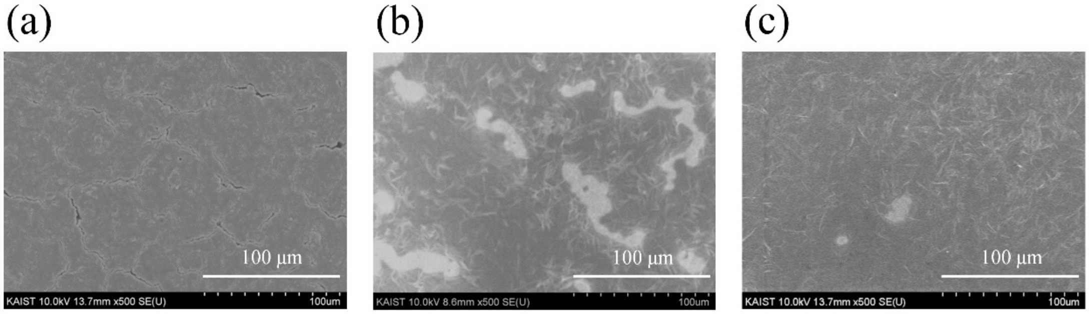

Fig. S2. SEM images of P(VDF-TrFE) films deposited on stainless steel substrates at 20 V for 2 min (a) before heat treatment, (b) after heat treatment and (c) after second deposition and heat treatment.

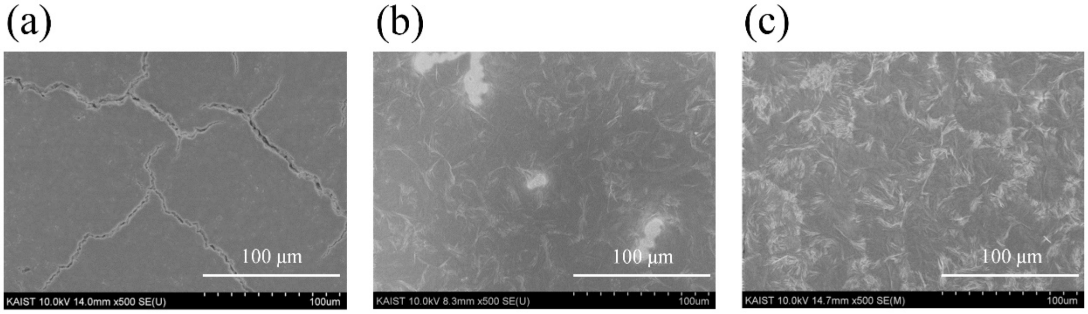

Fig. S3. SEM images of P(VDF-TrFE) films deposited on stainless steel substrates at 40 V for 2 min (a) before heat treatment, (b) after heat treatment and (c) after second deposition and heat treatment.

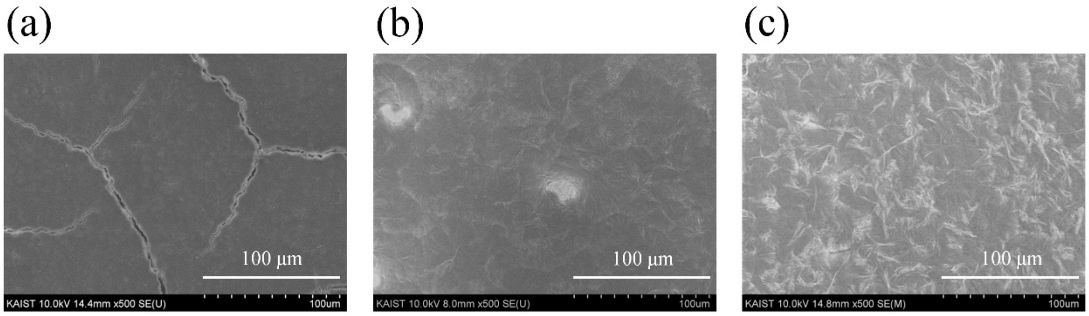

Fig. S4. SEM images of P(VDF-TrFE) films deposited on stainless steel substrates at 50 V for 2 min (a) before heat treatment, (b) after heat treatment and (c) after second deposition and heat treatment.

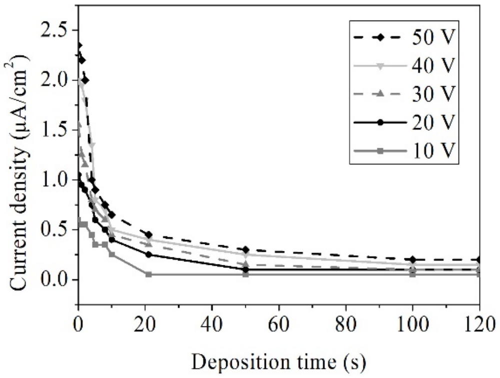

Fig. S5. Current density versus deposition time at different applied voltages during the 2nd EPD.

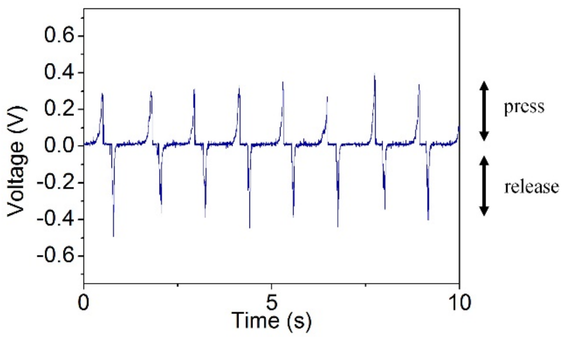

Fig S6. The output voltage of SPEH before the poling process.
